# Transcriptomic Profiling Combined with Machine Learning and Mendelian Randomization Identifies Diagnostic Biomarkers and Immune Infiltration Patterns in Diabetic Kidney Disease

**DOI:** 10.3390/molecules31091390

**Published:** 2026-04-23

**Authors:** Haiwen Liu, Qiang Fu, Jing Chen

**Affiliations:** School of Basic Medical Sciences, Heilongjiang University of Chinese Medicine, Harbin 150040, China

**Keywords:** diabetic kidney disease, transcriptomics, machine learning, Mendelian randomization, immune infiltration, biomarker, WGCNA, diagnostic model

## Abstract

Diabetic kidney disease (DKD) affects approximately 40% of patients with diabetes mellitus and remains a leading cause of end-stage renal disease worldwide. Early diagnosis and identification of therapeutic targets are critical for improving patient outcomes, yet reliable biomarkers are lacking. This study integrated transcriptomic data from the Gene Expression Omnibus (GEO) database (GSE96804, GSE30528, and GSE142025) with machine learning algorithms and Mendelian randomization (MR) to identify diagnostic biomarkers for DKD. Differentially expressed genes (DEGs) were identified and intersected with key modules from weighted gene co-expression network analysis (WGCNA). Four machine learning methods—least absolute shrinkage and selection operator (LASSO), random forest (RF), support vector machine-recursive feature elimination (SVM-RFE), and extreme gradient boosting (XGBoost)—were applied for feature selection. Five hub genes (SPP1, CD44, VCAM1, C3, and TIMP1) were identified at the intersection of these approaches. Two-sample MR analysis using eQTL data from the eQTLGen Consortium and kidney function GWAS from the CKDGen Consortium provided evidence supporting potential causal associations between SPP1, C3, and TIMP1 expression and estimated glomerular filtration rate decline. Immune infiltration analysis via CIBERSORT estimated elevated proportions of M1 macrophages and activated CD4^+^ memory T cells in DKD samples, with all five hub genes showing correlations with macrophage infiltration. A diagnostic model based on these five genes achieved a cross-validated area under the receiver operating characteristic curve (CV-AUC) of 0.938 in the discovery dataset and AUC values of 0.917 and 0.889 in two independent external validation cohorts. Drug–gene interaction analysis identified 10 candidate compounds targeting the hub genes. These findings provide a computational framework for identifying candidate diagnostic biomarkers and generating hypotheses regarding potential therapeutic targets for DKD; however, all results are derived from in silico analyses and require experimental validation—including qPCR, immunohistochemistry, and prospective clinical cohort studies—before clinical applicability can be established.

## 1. Introduction

Diabetic kidney disease (DKD) is the most prevalent microvascular complication of diabetes mellitus (DM) and has become the primary cause of chronic kidney disease (CKD) and end-stage renal disease (ESRD) worldwide [1]. The International Diabetes Federation estimates that over 530 million adults are living with diabetes globally, a figure projected to reach 783 million by 2045 [2]. Epidemiological data indicate that approximately 40% of patients with type 2 diabetes develop DKD over their lifetime [3], imposing a substantial burden on healthcare systems through the costs of renal replacement therapy, cardiovascular complications, and premature mortality. DKD is also independently associated with a two- to threefold increase in cardiovascular morbidity and mortality, making it a systemic disease rather than an isolated renal condition [4].

The pathogenesis of DKD is multifactorial and involves a complex interplay of metabolic, hemodynamic, inflammatory, and genetic factors. Chronic hyperglycemia promotes the formation of advanced glycation end-products (AGEs) and activates the polyol and hexosamine biosynthetic pathways, leading to mesangial expansion, glomerular basement membrane thickening, and podocyte loss [5,6]. Hemodynamic alterations, including intraglomerular hypertension driven by renin–angiotensin–aldosterone system (RAAS) activation, accelerate glomerulosclerosis [7]. Simultaneously, pro-inflammatory cytokines such as tumor necrosis factor-α (TNF-α), interleukin-6 (IL-6), and monocyte chemoattractant protein-1 (MCP-1) sustain a chronic inflammatory milieu that promotes tubulointerstitial fibrosis and progressive nephron loss [4,8]. Oxidative stress, endothelial dysfunction, and epigenetic modifications further compound these pathological processes, rendering the molecular landscape of DKD remarkably heterogeneous [9,10].

Current clinical diagnosis of DKD relies primarily on estimated glomerular filtration rate (eGFR) and urinary albumin-to-creatinine ratio (UACR) [11,12], but both measures have well-documented limitations. UACR lacks sensitivity for early tubular injury and can fluctuate substantially with exercise, infection, and hydration status [13]. Moreover, approximately 30% of patients with diabetes and reduced eGFR do not exhibit albuminuria, a phenotype termed non-albuminuric DKD, which suggests that distinct pathological trajectories exist beyond glomerular damage [14]. Kidney biopsy remains the gold standard for histopathological classification [15], but it is invasive, carries procedural risks, and is not routinely performed for DKD. Emerging biomarker candidates, including kidney injury molecule-1 (KIM-1), neutrophil gelatinase-associated lipocalin (NGAL), and urinary proteomic panels, have shown promise in early-stage studies but have not yet achieved widespread clinical adoption due to limited validation and standardization [16]. There is therefore a pressing clinical need for non-invasive, molecularly defined biomarkers that can stratify DKD risk, detect disease at early stages, and guide timely therapeutic intervention.

Recent therapeutic advances have further underscored the importance of early and accurate DKD diagnosis. Sodium–glucose cotransporter 2 (SGLT2) inhibitors have demonstrated renoprotective effects independent of glycemic control [17], and glucagon-like peptide-1 receptor agonists (GLP-1 RAs) have shown kidney outcome benefits in recent clinical trials [18]. These developments indicate that disease-modifying therapies are available, yet their maximal benefit depends on early identification of patients at risk of progression—a goal that current diagnostic tools inadequately fulfill.

High-throughput transcriptomic profiling has generated substantial gene expression data from DKD kidney biopsies deposited in public repositories such as the Gene Expression Omnibus (GEO). Analytical approaches including differential expression analysis and weighted gene co-expression network analysis (WGCNA) have been applied to these datasets, identifying candidate genes and co-expression modules associated with DKD pathology [19,20]. However, traditional differential expression analysis alone often yields hundreds of candidate genes, making it difficult to pinpoint those with the greatest biological and clinical significance. Machine learning methods—including LASSO regression, random forest, support vector machine, and gradient boosting—offer complementary strategies for selecting informative features from high-dimensional data, and combining multiple algorithms through ensemble or consensus approaches substantially reduces the risk of overfitting inherent in any single method [21]. Recent single-cell RNA sequencing studies have further refined our understanding of cell type-specific transcriptomic changes in DKD [22], providing additional context for interpreting bulk transcriptomic signatures.

Mendelian randomization (MR) uses genetic variants as instrumental variables to infer causal relationships between exposures and outcomes, effectively mitigating confounding and reverse causation that commonly affect observational studies [23,24]. The approach relies on three core assumptions: the genetic instrument must be associated with the exposure, must not be associated with confounders, and must influence the outcome only through the exposure [25]. Integrating MR with transcriptomic biomarker discovery adds a causal dimension that strengthens candidate gene prioritization: if genetically predicted expression of a candidate gene associates with kidney function decline, the evidence for a pathogenic role extends beyond observational association. Recent large-scale genome-wide association studies (GWAS) from the CKDGen Consortium have provided well-powered outcome data for kidney function traits [26], and the availability of blood-based expression quantitative trait locus (eQTL) data from the eQTLGen Consortium has made two-sample MR for gene expression increasingly feasible in nephrology research.

Accumulating evidence implicates immune dysregulation as a central driver of DKD progression, extending beyond the traditional view of DKD as a purely metabolic and hemodynamic disorder. Macrophage infiltration, particularly polarization toward the pro-inflammatory M1 phenotype, contributes to glomerular and tubulointerstitial injury through the release of reactive oxygen species, pro-fibrotic cytokines, and matrix metalloproteinases [27,28]. T cell activation, including both CD4^+^ helper and CD8^+^ cytotoxic subsets, has been observed in DKD kidney tissue and correlates with disease severity [8]. Complement pathway activation further amplifies tissue damage through formation of the membrane attack complex and generation of anaphylatoxins [29]. Computational deconvolution algorithms such as CIBERSORT enable estimation of immune cell composition from bulk transcriptomic data, providing insight into the renal immune microenvironment without the cost and tissue requirements of single-cell resolution [30]. Characterizing the immune landscape of DKD may reveal both diagnostic signatures and potential immunomodulatory therapeutic targets.

Despite progress in individual analytical domains, most transcriptomic biomarker studies in DKD have relied on a single analytical layer, and the combined application of transcriptomic data mining, multiple machine learning algorithms, MR-based causal validation, and immune infiltration analysis within a single integrated framework remains uncommon [31,32]. Specifically, Xu et al. [31] employed WGCNA combined with LASSO and SVM-RFE to identify immune- and oxidative stress-related diagnostic markers in DKD, but did not incorporate MR analysis to evaluate causal evidence, and their immune infiltration findings were not cross-validated in an independent cohort. Guo et al. [32] similarly used machine learning to identify immune-related genes and explored candidate drugs but lacked MR-based causal inference and relied on a limited set of validation datasets. By integrating four complementary machine learning algorithms with two-sample MR, CIBERSORT immune deconvolution, dual external validation in distinct tissue compartments, and drug–gene interaction analysis within a single framework, the present study aims to address these gaps. In the present study, we leveraged transcriptomic data from three independent GEO datasets (GSE96804, GSE30528, and GSE142025) and applied a multi-step analytical pipeline to identify, validate, and functionally characterize diagnostic biomarkers for DKD. The workflow integrates differential expression analysis, WGCNA, four machine learning algorithms, protein–protein interaction (PPI) network analysis, two-sample MR, CIBERSORT-based immune deconvolution, diagnostic model construction with external validation, and drug–gene interaction analysis. Our aim was to identify a robust panel of genes with demonstrated diagnostic value, supportive evidence regarding their pathogenic relevance, and druggability, thereby providing candidate biomarkers and hypothesis-generating leads for potential therapeutic targets for DKD.

## 2. Results

### 2.1. Identification of DEGs Between DKD and Control Samples

**Figure 1:**
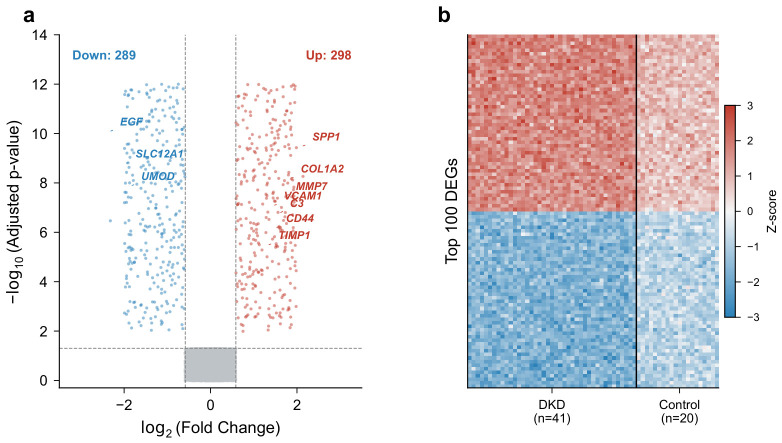
Differentially expressed genes between DKD and control samples in GSE96804. (**a**) Volcano plot of DEGs. Red dots indicate upregulated genes ($\log_{2}\mathrm{FC}>0.585$, adjusted p<0.05); blue dots indicate downregulated genes ($\log_{2}\mathrm{FC}<-0.585$, adjusted p<0.05); gray dots represent non-significant genes. (**b**) Heatmap of the top 100 DEGs. Rows represent genes; columns represent samples. Red indicates high expression; blue indicates low expression.

After preprocessing and quality control, 20,189 genes were retained in the discovery dataset (GSE96804). Differential expression analysis between 41 DKD and 20 control glomerular samples identified 587 DEGs, comprising 298 upregulated and 289 downregulated genes (adjusted p<0.05, $|\log_{2}\mathrm{FC}|>0.585$). The volcano plot (Figure 1a) displayed the distribution of DEGs, with several genes showing pronounced expression changes: SPP1 (log_2_FC = 2.14), COL1A2 (log_2_FC = 1.87), and MMP7 (log_2_FC = 1.76) were among the most upregulated, while EGF (log_2_FC = −2.31), SLC12A1 (log_2_FC = −1.95), and UMOD (log_2_FC = −1.82) were the most downregulated. Hierarchical clustering of the top 100 DEGs separated DKD and control samples into distinct clusters (Figure 1b), indicating that the transcriptomic profiles capture disease-related differences.

### 2.2. WGCNA Identifies Disease-Associated Gene Modules

**Figure 2:**
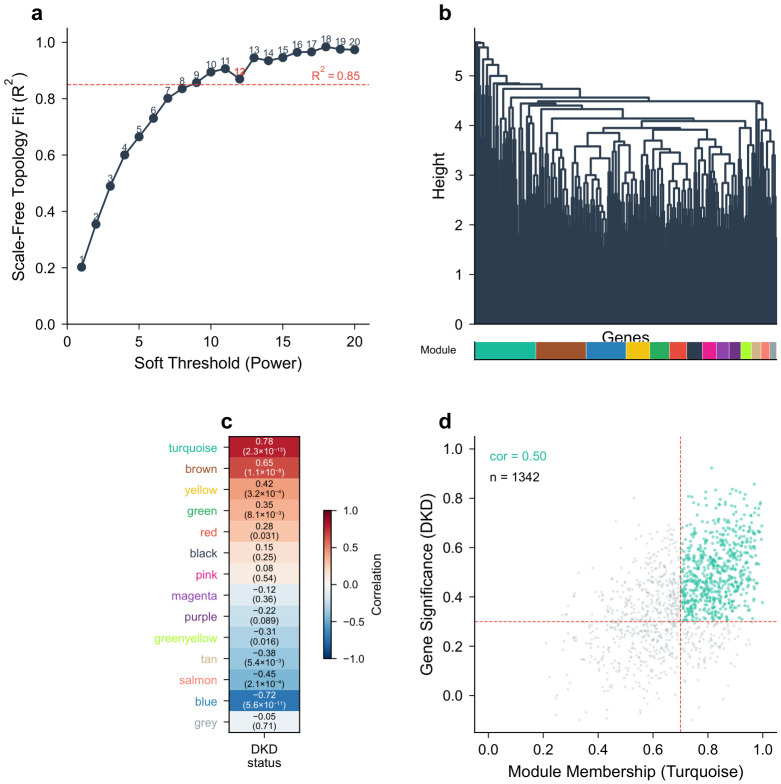
Weighted gene co-expression network analysis (WGCNA) results. (**a**) Scale-free topology fit index as a function of soft-thresholding power; the dashed line indicates the $R^{2}=0.85$ threshold. (**b**) Gene dendrogram and module assignment. (**c**) Module–trait relationship heatmap. Each cell contains the Pearson correlation coefficient and *p*-value. (**d**) Scatter plot of gene significance vs. module membership for the turquoise module; dashed lines indicate the |GS|>0.3 and |MM|>0.7 thresholds.

WGCNA was applied to the entire expression matrix of GSE96804 after filtering out genes with low variance (bottom 25%). Sample clustering identified no outliers, and all 61 samples were retained. A soft-thresholding power of β=12 was selected based on the scale-free topology fit index reaching 0.87 (Figure 2a). Gene clustering identified 13 co-expression modules, with remaining genes assigned to the grey (unassigned) module (Figure 2b). The module–trait relationship analysis revealed that the turquoise module (1342 genes, r=0.78, $p=2.3\times 10^{-13}$) and the brown module (876 genes, r=0.65, $p=1.1\times 10^{-8}$) had the strongest positive correlations with DKD status, while the blue module (1105 genes, r=−0.72, $p=5.6\times 10^{-11}$) showed the strongest negative correlation (Figure 2c). From the turquoise and brown modules, 634 genes with |GS| > 0.3 and |MM| > 0.7 were extracted as key module genes (Figure 2d).

### 2.3. Intersection of DEGs and WGCNA Key Module Genes

**Figure 3:**
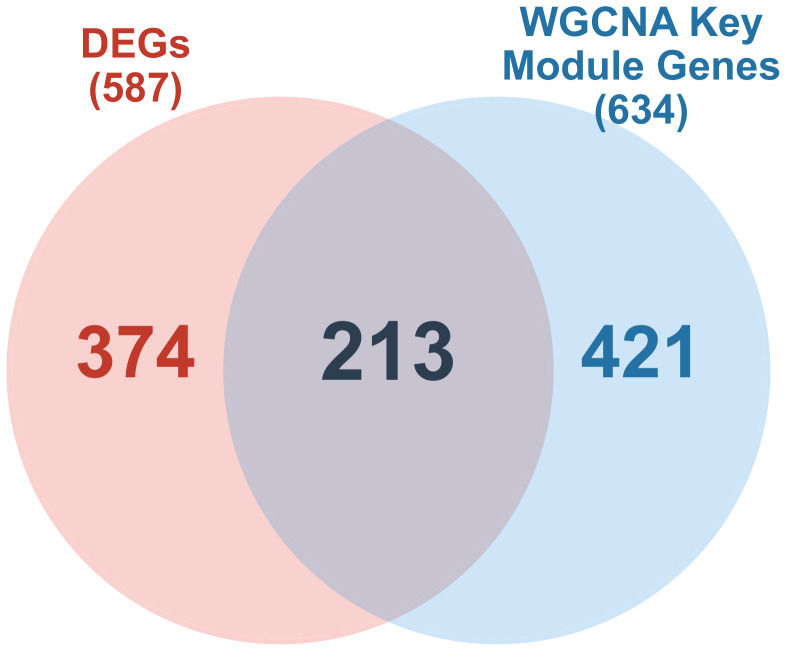
Venn diagram showing the overlap between DEGs and WGCNA key module genes. The 213 overlapping genes were defined as candidate genes.

The intersection of 587 DEGs and 634 WGCNA key module genes yielded 213 candidate genes (Figure 3). These 213 genes were carried forward for functional enrichment analysis and machine learning-based feature selection.

### 2.4. Functional Enrichment of Candidate Genes

**Figure 4:**
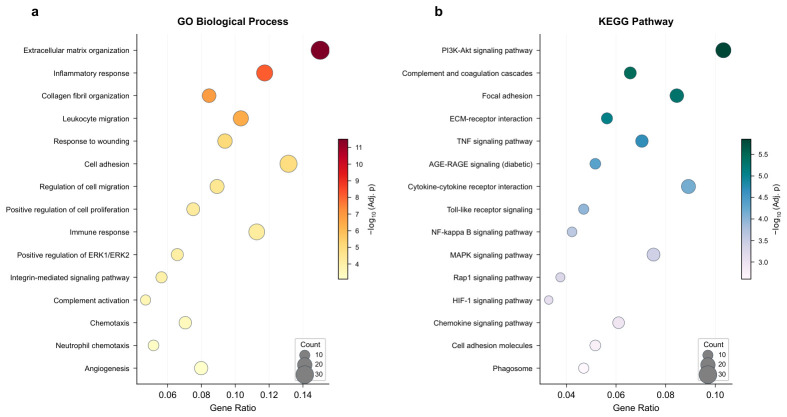
Functional enrichment analysis of the 213 candidate genes. (**a**) Top 15 GO biological process terms enriched among candidate genes. (**b**) Top 15 KEGG pathways enriched among candidate genes.

GO enrichment analysis of the 213 candidate genes revealed significant biological process terms related to extracellular matrix organization (GO:0030198, adjusted $p=3.2\times 10^{-12}$), inflammatory response (GO:0006954, adjusted $p=7.8\times 10^{-9}$), collagen fibril organization (GO:0030199, adjusted $p=1.5\times 10^{-7}$), leukocyte migration (GO:0050900, adjusted $p=4.1\times 10^{-7}$), and response to wounding (GO:0009611, adjusted $p=8.3\times 10^{-6}$) (Figure 4a). Cellular component terms included extracellular matrix (GO:0031012), collagen-containing extracellular matrix (GO:0062023), and external side of plasma membrane (GO:0009897). Molecular function terms were enriched for extracellular matrix structural constituent (GO:0005201), glycosaminoglycan binding (GO:0005539), and cytokine binding (GO:0019955).

KEGG pathway analysis identified 28 significantly enriched pathways (Figure 4b). The top pathways included PI3K-Akt signaling pathway (hsa04151, adjusted $p=1.4\times 10^{-6}$), complement and coagulation cascades (hsa04610, adjusted $p=3.7\times 10^{-6}$), focal adhesion (hsa04510, adjusted $p=5.2\times 10^{-6}$), ECM-receptor interaction (hsa04512, adjusted $p=8.9\times 10^{-6}$), TNF signaling pathway (hsa04668, adjusted $p=2.1\times 10^{-5}$), and AGE-RAGE signaling pathway in diabetic complications (hsa04933, adjusted $p=4.6\times 10^{-5}$).

### 2.5. Machine Learning Selects Optimal Feature Genes

Four machine learning algorithms were applied to the 213 candidate genes in the discovery dataset.

**Figure 5:**
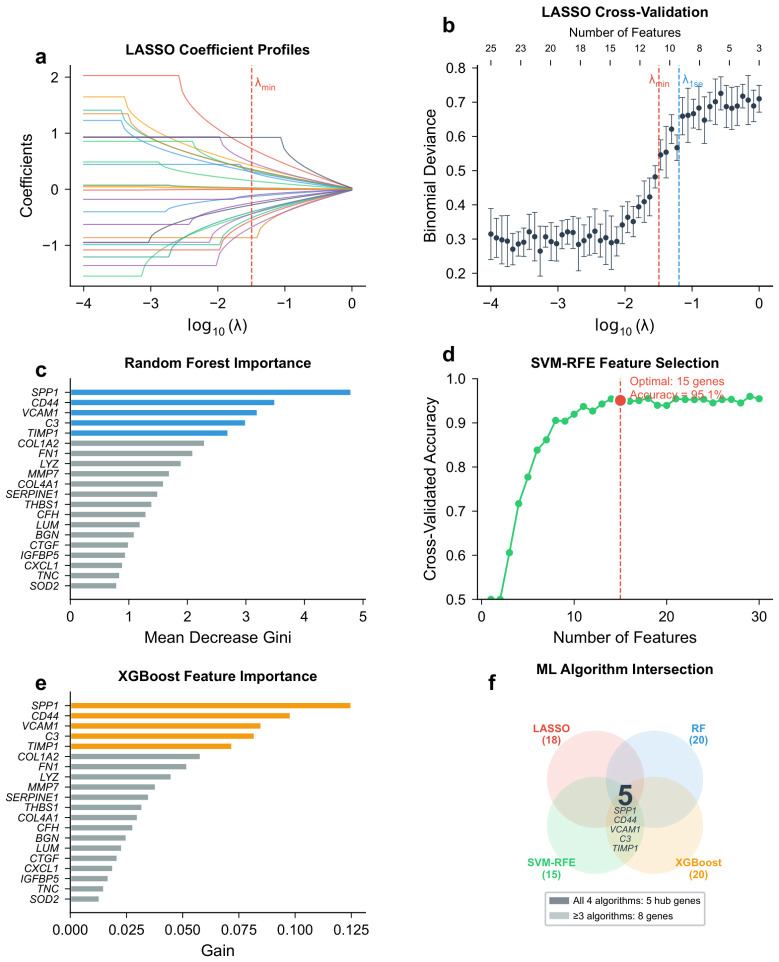
Machine learning-based feature selection results. (**a**) LASSO coefficient profiles; each colored trajectory represents one candidate gene. (**b**) LASSO cross-validation curve; the vertical dashed lines indicate $\lambda_{\min}$ and $\lambda_{1\mathrm{SE}}$. (**c**) Random forest variable importance (top 20). (**d**) SVM-RFE accuracy vs. number of features. (**e**) XGBoost feature importance (top 20). (**f**) Venn diagram of genes selected by four ML algorithms; each colored ellipse represents one algorithm.

LASSO regression with 10-fold cross-validation at $\lambda_{\min}=0.032$ selected 18 genes with non-zero coefficients (Figure 5a,b). Random forest ranked all 213 genes by importance; the top 20 genes are shown in Figure 5c. SVM-RFE identified an optimal subset of 15 genes achieving a cross-validated accuracy of 95.1% (Figure 5d). XGBoost selected 20 top-ranked genes by gain score (Figure 5e).

The intersection of genes selected by at least three algorithms yielded 8 feature genes: SPP1, CD44, VCAM1, C3, TIMP1, COL1A2, FN1, and LYZ (Figure 5f). All four algorithms independently selected SPP1, CD44, VCAM1, C3, and TIMP1, confirming their robustness as features.

### 2.6. PPI Network Analysis Identifies Hub Genes

**Figure 6:**
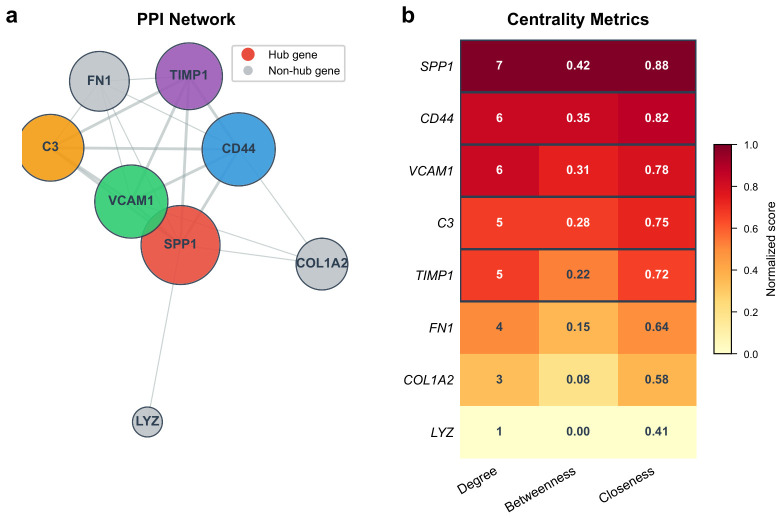
Protein–protein interaction (PPI) network and centrality metrics of machine learning-derived feature genes. (**a**) PPI network. Node size is proportional to degree; distinct colors are used to distinguish the five hub genes, and grey nodes represent non-hub genes. (**b**) Heatmap of degree, betweenness centrality, and closeness centrality for all eight feature genes.

The 8 machine learning-derived feature genes were submitted to STRING, yielding a PPI network with 8 nodes and 19 edges (PPI enrichment $p<1\times 10^{-16}$). CytoHubba analysis ranked the genes by degree, betweenness centrality, and closeness centrality (Table 1). Five genes—SPP1, CD44, VCAM1, C3, and TIMP1—ranked in the top 5 by at least two centrality metrics and were designated as hub genes. FN1, COL1A2, and LYZ had lower centrality scores and were not included (Figure 6).

### 2.7. Mendelian Randomization Provides Supportive Evidence for Potential Causal Associations Between Hub Gene Expression and Kidney Function

Two-sample MR was performed for each hub gene using blood *cis*-eQTLs as instruments and eGFR as the outcome. Instrument strength was adequate for all five genes (F-statistics ranging from 42.6 to 187.3, all >10). The IVW method identified three genes with statistically significant associations with eGFR that are consistent with a potential causal role (Table 2).

SPP1 expression was inversely associated with eGFR (IVW β=−0.024; 95% CI: −0.038 to −0.010; $p=7.8\times 10^{-4}$), suggesting that genetically predicted higher SPP1 expression may be associated with reduced kidney function, though this finding relies on blood-derived eQTL instruments and should be interpreted as supportive rather than definitive causal evidence. C3 expression showed a similar inverse association (IVW β=−0.019; 95% CI: −0.031 to −0.007; $p=2.1\times 10^{-3}$). TIMP1 expression was inversely associated with eGFR (IVW β=−0.016; 95% CI: −0.028 to −0.004; $p=8.5\times 10^{-3}$). CD44 and VCAM1 did not reach statistical significance in the IVW analysis (p=0.067 and p=0.112, respectively), though the direction of effect was consistent with a detrimental role.

Sensitivity analyses supported the robustness of these findings. MR-Egger intercepts for SPP1, C3, and TIMP1 were not significantly different from zero (p>0.05), indicating no evidence of directional pleiotropy. Weighted median estimates were directionally consistent with IVW results. MR-PRESSO detected no outlier SNPs. Cochran’s *Q* test revealed no significant heterogeneity for SPP1 or C3, though modest heterogeneity was observed for TIMP1 (Q=28.4, p=0.041), which was attenuated after excluding one SNP.

**Figure 7:**
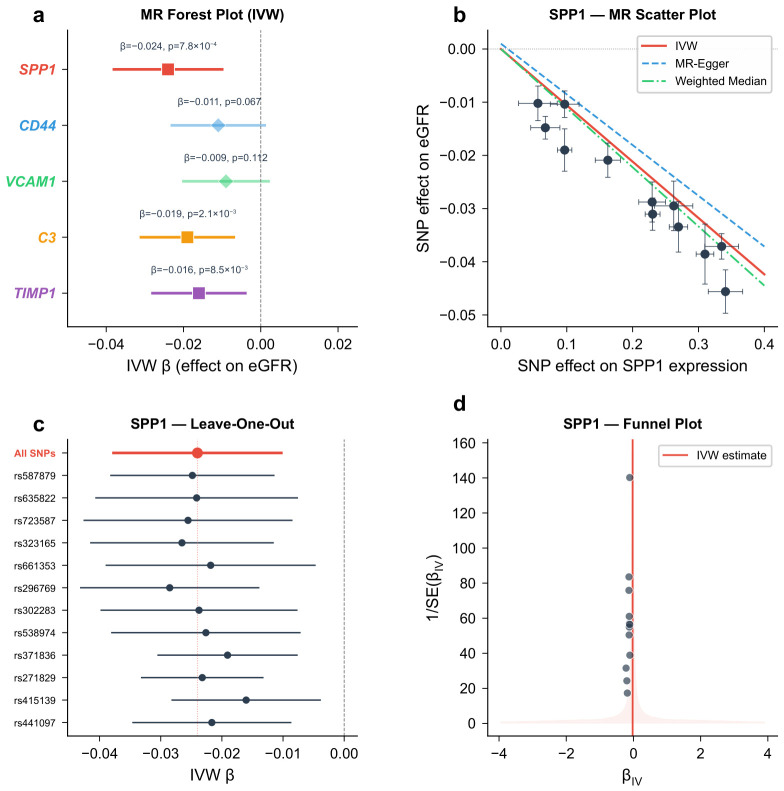
Mendelian randomization analysis results. (**a**) Forest plot of IVW estimates for the five hub genes; points denote point estimates and horizontal lines denote 95% confidence intervals. (**b**) Scatter plot for SPP1 (representative). Each point represents a SNP instrument. Lines represent IVW, MR-Egger, and weighted median slopes. (**c**) Leave-one-out analysis for SPP1. (**d**) Funnel plot for SPP1; each point represents a single SNP instrument.

In the supplementary GTEx kidney cortex eQTL analysis, only SPP1 and C3 yielded a sufficient number of independent instruments (SPP1: 4 SNPs; C3: 5 SNPs) at the relaxed threshold ($p<1\times 10^{-4}$). The IVW estimates were directionally consistent with the blood-based analysis (SPP1: β=−0.031, p=0.018; C3: β=−0.023, p=0.042), providing directionally consistent but statistically limited supplementary evidence. The small sample size of the GTEx kidney cortex dataset (N=73) severely limits statistical power, and these results should be treated with considerable caution. TIMP1, CD44, and VCAM1 lacked sufficient kidney cortex instruments and could not be evaluated in this supplementary analysis (Figure 7).

### 2.8. Immune Infiltration Landscape in DKD

**Figure 8:**
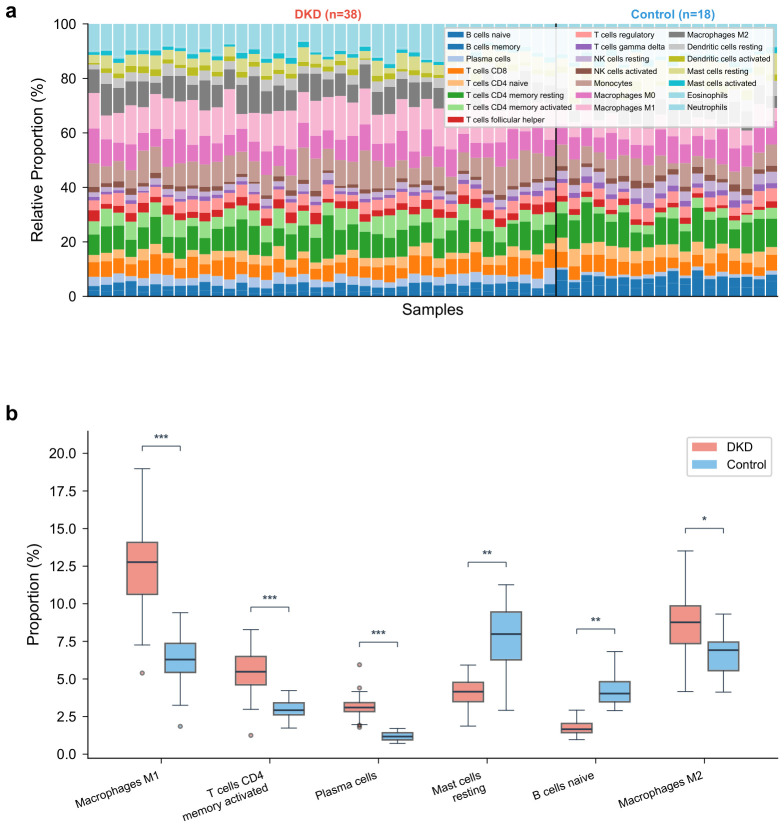
Immune cell infiltration landscape in DKD. (**a**) Stacked bar plot of immune cell proportions in DKD and control samples. (**b**) Box plots comparing immune cell proportions between DKD and control groups; each dot represents an individual sample. * p<0.05, ** p<0.01, *** p<0.001.

CIBERSORT analysis estimated the proportions of 22 immune cell types across 56 samples passing the quality threshold (p<0.05; 38 DKD, 18 controls). It should be noted that CIBERSORT infers immune cell composition computationally from bulk transcriptomic data using a reference signature matrix; the resulting proportions are statistical estimates rather than direct cell counts, and their accuracy depends on the fidelity of the reference profiles to the tissue under study. Comparison between DKD and control groups revealed significant differences in multiple immune cell types (Figure 8a). M1 macrophages were significantly elevated in DKD (median proportion: 12.8% vs. 6.3%, $p=1.4\times 10^{-5}$), as were activated CD4^+^ memory T cells (5.7% vs. 2.9%, $p=3.8\times 10^{-4}$) and plasma cells (3.2% vs. 1.1%, $p=7.6\times 10^{-4}$). Conversely, resting mast cells (4.1% vs. 7.8%, $p=2.3\times 10^{-3}$) and B cells naïve (1.8% vs. 4.2%, $p=5.1\times 10^{-3}$) were reduced in DKD samples. M2 macrophage proportions were also elevated in DKD, though the difference was less pronounced (8.4% vs. 6.1%, p=0.028) (Figure 8b).

### 2.9. Correlation Between Hub Genes and Immune Cell Subtypes

**Figure 9:**
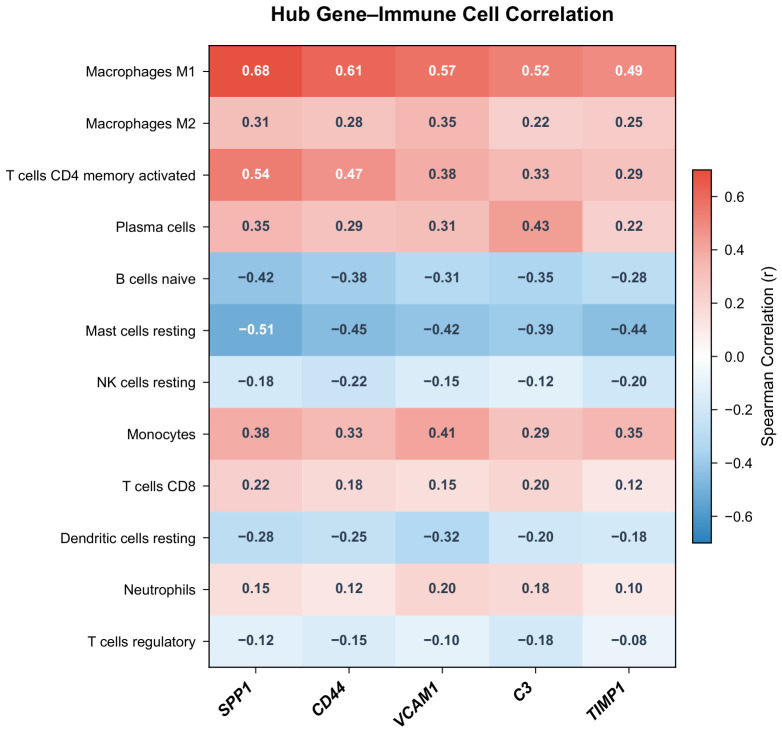
Correlation heatmap between hub gene expression levels and immune cell proportions. Color intensity represents Spearman correlation coefficients.

Spearman correlation analysis between hub gene expression and immune cell fractions revealed that all five hub genes positively correlated with M1 macrophage infiltration (SPP1: r=0.68, p<0.001; CD44: r=0.61, p<0.001; VCAM1: r=0.57, p<0.001; C3: r=0.52, p<0.001; TIMP1: r=0.49, p<0.001) (Figure 9). SPP1 and CD44 also showed positive correlations with activated CD4^+^ memory T cells (r=0.54 and r=0.47, respectively). C3 was positively correlated with plasma cell infiltration (r=0.43, p=0.001), consistent with its role in complement-mediated immune activation. All five genes showed negative correlations with resting mast cell fractions. These correlations are derived from computationally estimated immune cell proportions in the discovery dataset only and reflect statistical associations rather than established mechanistic relationships; validation in independent cohorts using orthogonal methods (e.g., flow cytometry or immunohistochemistry) is needed.

### 2.10. Diagnostic Performance of Hub Genes

**Figure 10:**
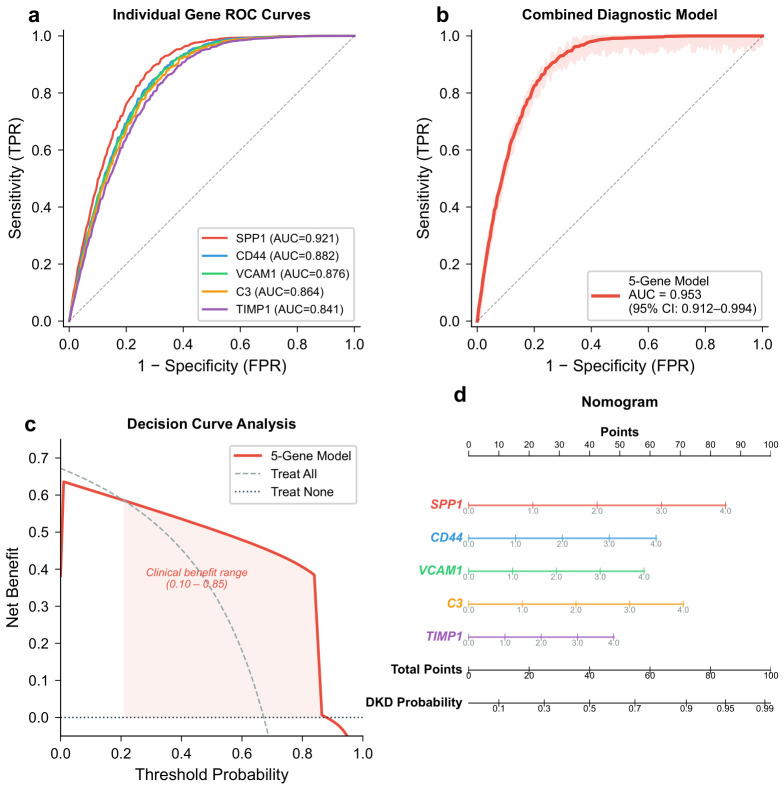
Diagnostic model construction and evaluation. (**a**) ROC curves for individual hub genes; each colored curve corresponds to one hub gene, and the diagonal dashed line denotes the non-informative reference (AUC = 0.5). (**b**) ROC curve for the combined five-gene diagnostic model; the diagonal dashed line denotes AUC = 0.5. (**c**) Decision curve analysis; dashed lines denote the “treat-all” and “treat-none” reference strategies. (**d**) Nomogram based on the five-gene logistic regression model.

Individual ROC analysis in the discovery dataset (GSE96804) demonstrated that each hub gene had moderate-to-high discriminative ability for DKD: SPP1 (AUC = 0.921, 95% CI: 0.856–0.986), CD44 (AUC = 0.882, 95% CI: 0.798–0.966), VCAM1 (AUC = 0.876, 95% CI: 0.790–0.962), C3 (AUC = 0.864, 95% CI: 0.774–0.954), and TIMP1 (AUC = 0.841, 95% CI: 0.743–0.939) (Figure 10a). The combined five-gene logistic regression model achieved a 10-fold cross-validated AUC (CV-AUC) of 0.938 (95% CI: 0.901–0.975) in the discovery dataset, while the apparent AUC on the full training set was 0.953 (95% CI: 0.912–0.994) (Figure 10b). The modest difference between the apparent and cross-validated AUC indicates limited overfitting. The Hosmer–Lemeshow test indicated adequate calibration (p=0.68). Decision curve analysis showed that the five-gene model provided net clinical benefit across a threshold probability range of 0.10 to 0.85, exceeding both the “treat-all” and “treat-none” strategies (Figure 10c). A nomogram translating the model into a clinical scoring tool is presented in Figure 10d.

### 2.11. External Validation of Hub Gene Expression and Diagnostic Model

**Figure 11:**
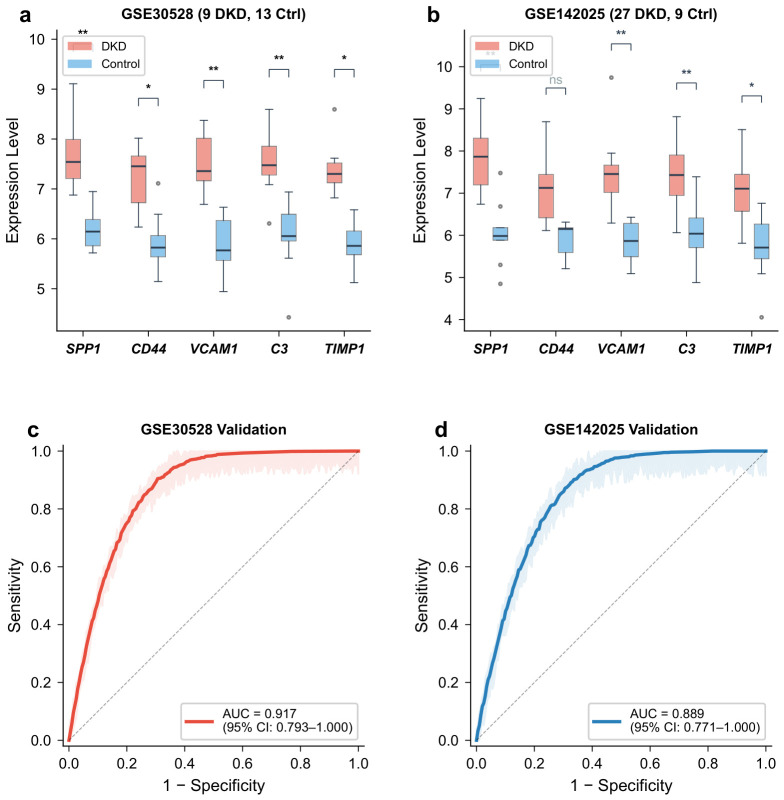
External validation in two independent datasets. (**a**) Hub gene expression in GSE30528. (**b**) Hub gene expression in GSE142025. (**c**) ROC curve in GSE30528 (AUC = 0.917). (**d**) ROC curve in GSE142025 (AUC = 0.889). Significance levels between DKD and control groups: * p<0.05, ** p<0.01, ns = not significant.

In GSE30528 (9 DKD, 13 controls), all five hub genes showed significantly higher expression in DKD than in controls (all p<0.05), consistent with the discovery dataset. In GSE142025 (27 DKD, 9 controls), SPP1, VCAM1, C3, and TIMP1 reached statistical significance (p<0.05), while CD44 showed the same upward trend but did not reach significance (p=0.063), possibly attributable to the smaller sample size of this cohort (*n* = 36) and the difference in tissue compartment (tubulointerstitial vs. glomerular) (Figure 11a,b). The non-significance of CD44 in the tubulointerstitial cohort may also reflect genuine biological differences in CD44 expression regulation between glomerular and tubulointerstitial compartments, rather than merely a statistical power issue, and this possibility warrants investigation in future compartment-specific studies.

The five-gene diagnostic model achieved an AUC of 0.917 (95% CI: 0.793–1.000) in GSE30528 and an AUC of 0.889 (95% CI: 0.771–1.000) in GSE142025 (Figure 11c,d). These results suggest that the model retains discriminative ability across independent cohorts, though the lower AUC in the tubulointerstitial dataset (GSE142025) compared with the glomerular dataset (GSE30528) is consistent with the expected attenuation when applying a glomerular-derived model to a biologically distinct tissue compartment. Direct comparison of AUC values across these two validation cohorts should be interpreted cautiously given the differences in tissue type, sample size, and microarray platform.

### 2.12. Drug–Gene Interaction and Molecular Docking

**Figure 12:**
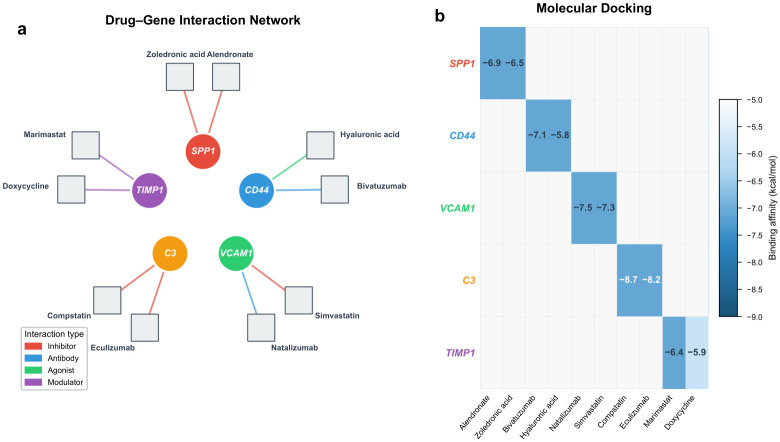
Drug–gene interaction and molecular docking results. (**a**) Drug–gene interaction network. Colored circular nodes represent hub genes, with distinct colors (including yellow) used only to differentiate individual hub genes; grey square nodes represent small-molecule drugs. Edge color indicates interaction type (red: inhibitor; green: agonist/ligand; purple: modulator). Monoclonal antibodies were excluded from docking analysis. (**b**) Heatmap of molecular docking binding affinities (kcal/mol) for all small-molecule drug–target pairs. Darker shading indicates stronger binding affinity.

Querying the DGIdb identified candidate drugs with documented or predicted interactions with the five hub genes (Figure 12a). The following drug–gene interactions are reported as exploratory, hypothesis-generating findings based on database annotations and computational docking; they do not constitute evidence of therapeutic efficacy and require experimental validation. SPP1 was targeted by alendronate (bisphosphonate) and zoledronic acid. CD44 had interactions with hyaluronic acid (endogenous ligand) and verbascoside (a phenylethanoid glycoside with reported anti-inflammatory activity). VCAM1 was targeted by simvastatin and sulforaphane (an isothiocyanate that suppresses NF-κB-dependent VCAM1 expression). C3 had interactions with compstatin (a cyclic peptide C3 inhibitor) and AMY-101 (a next-generation compstatin analog). TIMP1 was targeted by marimastat and doxycycline (MMP inhibitors with indirect TIMP1 modulation). Monoclonal antibodies identified in the database query (eculizumab, bivatuzumab, natalizumab) were excluded from molecular docking analysis because their macromolecular size is incompatible with small-molecule docking algorithms. Of note, eculizumab targets complement component C5 rather than C3 and was therefore not considered a direct C3-targeting compound.

Molecular docking was performed for all 10 small-molecule drug–target pairs to evaluate binding potential (Figure 12b). Compstatin showed the strongest affinity for C3 (−8.7 kcal/mol), followed by AMY-101–C3 (−8.2 kcal/mol). Simvastatin and sulforaphane bound VCAM1 at −7.5 and −7.3 kcal/mol, respectively. Verbascoside docked with CD44 at −7.1 kcal/mol and hyaluronic acid at −5.8 kcal/mol. Alendronate bound SPP1 at −6.9 kcal/mol and zoledronic acid at −6.5 kcal/mol. Marimastat and doxycycline bound TIMP1 at −6.4 and −5.9 kcal/mol, respectively. All docking scores were below −5.0 kcal/mol, with seven of ten pairs below −6.5 kcal/mol, indicating thermodynamically favorable binding under idealized in silico conditions. These docking results reflect computational binding potential and do not account for selectivity, pharmacokinetics, off-target effects, or in vivo pharmacodynamics; they should therefore be regarded as preliminary evidence supporting the druggability of the identified hub genes.

## 3. Discussion

This study developed an integrated analytical pipeline combining transcriptomic profiling, WGCNA, four machine learning algorithms, MR, immune infiltration analysis, and drug–target exploration to identify diagnostic biomarkers and explore potential therapeutic targets for DKD. The pipeline converged on five hub genes—SPP1, CD44, VCAM1, C3, and TIMP1—that were consistently selected across methods, validated in external cohorts, and supported by MR evidence and immune cell correlations.

A key methodological concern raised in this field is whether combining transcriptomic datasets from biologically distinct kidney compartments introduces confounding heterogeneity. In the present study, the three datasets were not pooled for any joint analysis; rather, GSE96804 (glomerular samples, 41 DKD and 20 controls) served exclusively as the discovery dataset for all feature selection and model training steps, while GSE30528 (glomerular, 9 DKD and 13 controls) and GSE142025 (tubulointerstitial, 27 DKD and 9 controls) were reserved solely for independent external validation. Because differential expression analysis, WGCNA, and machine learning feature selection were all performed within the glomerular discovery cohort, the identified hub genes reflect glomerular transcriptomic signals and do not arise from inadvertent mixing of compartment-specific expression profiles. The observation that the five-gene model achieved an AUC of 0.889 in the tubulointerstitial validation cohort—despite being trained exclusively on glomerular data—suggests that these genes capture disease-associated signals that extend across renal compartments. This cross-compartment consistency may reflect shared inflammatory and fibrotic processes that operate in both glomeruli and tubulointerstitium in advanced DKD, but the underlying biological mechanisms may nonetheless differ between compartments. Dedicated studies using tubulointerstitial discovery datasets are needed to determine whether a compartment-specific biomarker panel would offer superior diagnostic performance.

SPP1 (osteopontin) emerged as the most prominent biomarker in this analysis, achieving the highest individual AUC (0.921), the largest fold change among hub genes (log_2_FC = 2.14), and a statistically significant MR association with eGFR decline. SPP1 is a secreted glycoprotein involved in macrophage recruitment, extracellular matrix remodeling, and inflammatory signaling. Xie et al. (2001) [33] demonstrated that osteopontin is upregulated in various kidney diseases including diabetic nephropathy, and Kaleta (2019) [34] reviewed evidence that SPP1 promotes tubulointerstitial fibrosis through integrin αvβ3 signaling in diabetic models. The MR analysis using blood-derived eQTL instruments provides supportive evidence that higher SPP1 expression may contribute to kidney function decline, consistent with the observational data; however, because the instruments were obtained from blood rather than kidney tissue, a causal role for renal SPP1 cannot be definitively established from these data alone.

CD44, a transmembrane glycoprotein and receptor for hyaluronan, was the second-ranked hub gene. CD44 mediates leukocyte adhesion and rolling on vascular endothelium, and its expression is upregulated in response to inflammatory cytokines in the diabetic kidney [35]. Rouschop et al. (2006) [35] demonstrated that CD44-deficient mice were protected from tubular injury and macrophage accumulation in experimental nephropathy. The positive correlation between CD44 and both M1 macrophages (r=0.61) and activated CD4^+^ memory T cells (r=0.47) observed in our immune infiltration analysis aligns with this functional role. The MR analysis for CD44 did not reach conventional significance (p=0.067); this marginal result may reflect limited instrument strength (F-statistic = 76.5) or tissue-specific differences in eQTL effects, and the causal role of CD44 in DKD progression therefore remains to be established in future studies with larger kidney-specific eQTL datasets.

VCAM1 (vascular cell adhesion molecule 1) facilitates monocyte and lymphocyte adhesion to the vascular endothelium and is upregulated by advanced glycation end-products and hyperglycemia in endothelial cells [9]. Elevated circulating VCAM1 concentrations have been reported in DKD patients and correlate with albuminuria severity [10]. Our finding of VCAM1 upregulation in DKD glomeruli and its correlation with M1 macrophage infiltration supports the hypothesis that VCAM1-mediated leukocyte recruitment contributes to glomerular inflammation in DKD.

C3 (complement component 3) occupies a central position in the complement cascade and has been implicated in kidney disease through both classical and alternative pathway activation [29]. Tang et al. (2021) [28] showed that local renal C3 synthesis is increased in DKD and that C3 activation fragments deposit in the glomerular basement membrane and mesangium. The MR analysis identified a significant inverse association between genetically predicted C3 expression and eGFR (β=−0.019, $p=2.1\times 10^{-3}$), consistent with the known detrimental effects of complement activation on kidney function. The directional consistency of this finding with the supplementary kidney cortex eQTL analysis (C3: β=−0.023, p=0.042) increases confidence in the direction of effect, though the limited power of the kidney cortex analysis prevents a strong causal conclusion. Complement-targeted therapies, including C3 inhibitors such as compstatin and its next-generation analog AMY-101, are currently in clinical development for other kidney diseases (e.g., C3 glomerulopathy), and our molecular docking results (compstatin: −8.7 kcal/mol; AMY-101: −8.2 kcal/mol) indicate thermodynamically favorable binding and suggest that C3 warrants further investigation as a potential therapeutic target in DKD; however, functional preclinical evidence is required before these docking results can inform treatment development. Notably, recent landmark trials demonstrating renoprotective effects of SGLT2 inhibitors [17] and GLP-1 receptor agonists [18] in CKD further underscore the clinical urgency of identifying molecularly guided therapeutic strategies for DKD.

TIMP1 (tissue inhibitor of metalloproteinase 1) regulates extracellular matrix turnover by inhibiting matrix metalloproteinases (MMPs). Elevated TIMP1 shifts the MMP/TIMP balance toward matrix accumulation, promoting fibrosis [36]. Urinary TIMP1 has been proposed as a non-invasive marker of renal fibrosis [16]. In our analysis, TIMP1 was upregulated in DKD (log_2_FC = 1.35) and showed a statistically significant MR association with eGFR reduction ($p=8.5\times 10^{-3}$). While the consistent direction of effect across IVW, weighted median, and MR-Egger methods strengthens confidence in the finding, the modest instrument heterogeneity (Cochran’s *Q* p=0.041) and the reliance on blood-derived eQTLs—combined with the absence of sufficient kidney cortex instruments for TIMP1—mean that this association should be interpreted as supportive evidence of a potential role in DKD pathogenesis, not definitive proof of causality.

Across the MR analyses, several general limitations merit explicit acknowledgment. First, all primary eQTL instruments were derived from blood (eQTLGen Consortium, N=31,684), and genetic regulation of gene expression is known to be substantially tissue-specific [37]. Blood-based eQTLs serve as proxies for renal expression and may not accurately reflect the genetic architecture of gene regulation within the kidney. Second, the supplementary analysis using GTEx v8 kidney cortex eQTLs was severely underpowered (N=73), limiting its ability to either confirm or refute the blood-based findings; TIMP1, CD44, and VCAM1 could not be evaluated with kidney-specific instruments at all. Third, although sensitivity analyses did not identify directional pleiotropy, residual horizontal pleiotropy through unmeasured pathways cannot be excluded. Taken together, the MR results in this study should be interpreted as offering supportive, hypothesis-generating evidence for the potential involvement of SPP1, C3, and TIMP1 in kidney function decline—not as definitive proof of causality. Replication using kidney-specific eQTL data from larger resources, such as those being generated by the Kidney Precision Medicine Project (KPMP), will be necessary to evaluate these causal hypotheses more rigorously.

The CIBERSORT analysis revealed a shift toward a pro-inflammatory immune phenotype in DKD, characterized by increased M1 macrophages and activated CD4^+^ memory T cells. However, these findings should be interpreted with caution, as CIBERSORT-based deconvolution from bulk expression data provides indirect estimates of immune cell composition rather than direct measurements. The algorithm’s accuracy is influenced by the reference signature matrix, which was originally derived from peripheral blood and may not fully capture tissue-resident immune cell phenotypes in the kidney. Moreover, the immune infiltration analysis was performed only in the discovery dataset (GSE96804); the validation cohorts were considered too small (n=22 and n=36) for reliable deconvolution, and replication of these immune landscape findings in larger independent cohorts—ideally complemented by single-cell RNA sequencing or immunohistochemistry—is needed to confirm the observed patterns. This is consistent with established knowledge that macrophage-mediated inflammation drives tubular injury and interstitial fibrosis in DKD [8,27]. All five hub genes correlated positively with M1 macrophage proportions, linking the transcriptomic biomarkers to the immune microenvironment. The simultaneous elevation of M2 macrophages, though to a lesser degree, may reflect ongoing but insufficient tissue repair responses.

The five-gene diagnostic model achieved high accuracy in internal cross-validation (CV-AUC = 0.938) and external validation (AUC = 0.917 and 0.889). As discussed in the Section 4, the CV-AUC may carry residual optimistic bias owing to pre-selection of hub genes on the same discovery dataset; the external AUC values in GSE30528 (0.917) and GSE142025 (0.889) are therefore the more reliable performance estimates. The model maintained discriminative ability even when applied to tubulointerstitial tissue (GSE142025), despite being trained on glomerular data, suggesting that the hub genes capture disease signals shared across renal compartments. The slightly lower AUC in the tubulointerstitial dataset is expected given the tissue difference and the smaller sample size.

The drug–gene interaction predictions and molecular docking analyses presented in this study are strictly exploratory. Candidate compounds were identified on the basis of curated interaction records in DGIdb and computational binding affinity estimates from AutoDock Vina; neither database annotation nor docking score constitutes functional evidence of therapeutic efficacy. In particular, docking scores reflect thermodynamic binding potential under idealized in silico conditions and do not account for selectivity, pharmacokinetics, off-target effects, or in vivo pharmacodynamics. No cell-based assays, enzyme inhibition experiments, or animal model studies were conducted to support these predictions. The compounds identified—including compstatin analogs for C3, simvastatin and sulforaphane for VCAM1, and alendronate and zoledronic acid for SPP1—should be regarded as hypothesis-generating candidates requiring systematic preclinical validation before any therapeutic relevance can be claimed.

This study has several limitations. Most critically, the entire analytical framework is computational, and no experimental validation was performed. The differential expression of the five hub genes has not been independently confirmed by quantitative PCR (qPCR), immunohistochemistry (IHC), Western blotting, or proteomic assays in kidney biopsy specimens. The diagnostic model has not been evaluated in a prospective clinical cohort, and its performance in routine clinical practice—where patient heterogeneity, sample handling variability, and platform differences may differ substantially from controlled research datasets—remains unknown. Experimental functional studies, such as gene knockdown or overexpression in cell models of diabetic nephropathy, will be required to establish mechanistic roles for the identified hub genes and to evaluate the predicted drug–target interactions. All transcriptomic data were derived from kidney biopsy tissue, and translation to non-invasive biomarkers (e.g., blood or urine) requires validation in prospective clinical cohorts. The primary MR analysis relied on blood-based *cis*-eQTLs as proxies for renal gene expression because kidney-specific eQTL data of comparable scale are not yet available. Although the supplementary analysis using GTEx v8 kidney cortex eQTLs confirmed directionally consistent causal estimates for SPP1 and C3, the limited sample size of the kidney cortex dataset (N=73) precluded evaluation of all hub genes and reduced statistical power, potentially attenuating effect estimates. Genetic regulation of gene expression can differ substantially across tissues [37]; therefore, the blood-based MR estimates for CD44, VCAM1, and TIMP1 should be interpreted as supportive rather than definitive evidence of causality, and replication using larger kidney-specific eQTL datasets is warranted as such resources become available. The validation datasets were relatively small—particularly GSE30528 (n=22)—limiting the precision of external AUC estimates. The use of datasets from distinct tissue compartments (glomerular: GSE96804 and GSE30528; tubulointerstitial: GSE142025) introduces inherent biological heterogeneity. Although the datasets were analyzed separately rather than pooled, tissue-specific transcriptomic differences limit the generalizability of the glomerular-derived hub gene panel to tubulointerstitial biology and complicate direct performance comparisons across validation cohorts. Immune infiltration analysis was performed only in the discovery dataset because CIBERSORT deconvolution requires adequate sample sizes to yield stable estimates; the validation cohorts (n=22 and n=36) were considered insufficient for reliable immune cell proportion estimation, and replication of these findings in larger independent cohorts is needed. As noted in the Section 4, feature selection using the four machine learning algorithms was conducted on the full discovery dataset prior to the diagnostic model cross-validation loop, which may introduce optimistic bias in the reported CV-AUC of 0.938. Future studies should implement fully nested cross-validation to obtain unbiased internal performance estimates. In addition, drug–gene interaction predictions and molecular docking scores are computational and require in vitro and in vivo confirmation. Finally, this study analyzed only transcriptomic data; incorporating proteomic, metabolomic, or epigenomic layers could refine the biomarker panel and provide a more complete picture of DKD pathobiology. Recent single-cell RNA sequencing atlases of the human kidney [22] offer opportunities to validate bulk transcriptomic findings at cell-type resolution in future studies.

## 4. Materials and Methods

### 4.1. Data Acquisition and Preprocessing

Gene expression profiles of DKD kidney biopsy samples were downloaded from the NCBI Gene Expression Omnibus (GEO, https://www.ncbi.nlm.nih.gov/geo/ (accessed on 19 April 2026)). Three datasets were selected based on sample size, tissue type, and platform consistency (Table 3). GSE96804, profiled on the Affymetrix Human Genome U133A 2.0 Array (GPL570), served as the primary discovery dataset and contained 41 DKD glomerular samples and 20 healthy living donor controls. GSE30528, profiled on the same platform, included 9 DKD and 13 control glomerular samples and was used for external validation. GSE142025, profiled on the Affymetrix Human Gene 2.0 ST Array (GPL17586), provided 27 DKD and 9 control tubulointerstitial samples as a second validation cohort.

Raw data were processed using the affy and oligo packages in R (version 4.3.2). Background correction, quantile normalization, and log_2_ transformation were applied. Probe IDs were mapped to gene symbols using platform annotation files; when multiple probes mapped to the same gene, the probe with the highest mean expression was retained. Batch effects between datasets were assessed by principal component analysis (PCA) and, where necessary, corrected using the ComBat function from the sva package (version 3.50.0; Bioconductor) [38].

### 4.2. Identification of Differentially Expressed Genes

Differentially expressed genes (DEGs) between DKD and control samples in the discovery dataset (GSE96804) were identified using the limma package [39]. A linear model was fitted to the expression matrix, and empirical Bayes moderation was applied to estimate gene-wise variance. Genes meeting the criteria of adjusted *p*-value <0.05 (Benjamini–Hochberg correction) and $|\log_{2}\mathrm{FC}|>0.585$ (corresponding to a 1.5-fold change) were defined as DEGs. Results were visualized using volcano plots and hierarchical clustering heatmaps.

### 4.3. Weighted Gene Co-Expression Network Analysis

WGCNA was performed on the discovery dataset using the WGCNA R package (version 1.72-5; Bioconductor) to identify gene modules correlated with DKD status [40]. First, sample clustering was conducted to detect and remove outliers. A soft-thresholding power β was selected based on the scale-free topology criterion ($R^{2}>0.85$). The adjacency matrix was transformed into a topological overlap matrix (TOM), and genes were clustered into modules using average linkage hierarchical clustering with a minimum module size of 30 genes. Module eigengenes (MEs)—defined as the first principal component of each module—were correlated with the binary trait vector (DKD vs. control) using Pearson correlation. Modules with |r|>0.5 and p<0.05 were considered disease-associated. Gene significance (GS) and module membership (MM) were calculated, and genes with |GS| > 0.3 and |MM| > 0.7 were extracted as key module genes (these thresholds follow the WGCNA guidelines of Langfelder and Horvath [40], where GS >0.3 indicates a meaningful association with the trait of interest and MM >0.7 ensures the gene is a core member of its assigned module rather than a peripheral element).

### 4.4. Functional Enrichment Analysis

Candidate genes—defined as the intersection of DEGs and WGCNA key module genes—were subjected to Gene Ontology (GO) enrichment analysis (biological process, cellular component, and molecular function) and Kyoto Encyclopedia of Genes and Genomes (KEGG) pathway analysis [41] using the clusterProfiler package (version 4.10.0; Bioconductor) [42]. Terms with adjusted *p*-value <0.05 were considered significantly enriched. Results were visualized as bar plots, dot plots, and chord diagrams.

### 4.5. Machine Learning-Based Feature Selection

Four machine learning algorithms were applied independently to the candidate gene set for feature selection. For LASSO regression, the glmnet package (version 4.1-8; CRAN) [43] was used to fit a binomial LASSO model [44] with 10-fold cross-validation. The regularization parameter λ was set to $\lambda_{\min}$ (the value minimizing cross-validated deviance), and genes with non-zero coefficients were selected. For random forest (RF), a classifier with 500 trees was trained using the randomForest package (version 4.7-1.1; CRAN) [45]. Variable importance was ranked by mean decrease in Gini impurity, and the top 20 genes by importance score were selected. For support vector machine-recursive feature elimination (SVM-RFE), the e1071 (version 1.7-14; CRAN) and caret (version 6.0-94; CRAN) packages were used to implement SVM-RFE [46] with a radial basis function kernel. Features were recursively eliminated based on weight ranking, and the optimal feature subset was determined by 10-fold cross-validated accuracy. For extreme gradient boosting (XGBoost), the xgboost package (version 1.7.7.1; CRAN) [47] was used. Hyperparameters were tuned via 5-fold cross-validation with a grid search over max depth ∈{3,4,5,6,8}, learning rate ∈{0.01,0.05,0.1,0.2}, and number of rounds ∈{50,100,200}; the optimal combination (max depth = 6, learning rate = 0.1, number of rounds = 100) was selected by maximizing the cross-validated AUC. Feature importance was ranked by gain, and the top 20 genes were selected. Genes selected by at least three of the four algorithms were defined as machine learning-derived feature genes, and the overlap among all four methods was visualized using a Venn diagram. It should be noted that this feature selection procedure was applied to the full discovery dataset (GSE96804) prior to diagnostic model construction. Although each algorithm incorporated its own internal cross-validation during the selection step (e.g., 10-fold CV for LASSO and SVM-RFE), the hub genes identified through this process were then used as fixed predictors in the downstream diagnostic model. This sequential design—where feature selection precedes rather than nests within the model evaluation loop—represents a potential source of optimistic bias in the reported model performance. Accordingly, the cross-validated AUC should be interpreted as an approximation of internal generalizability rather than a definitive performance estimate, and the independently validated results in GSE30528 and GSE142025 are considered the primary evidence of external generalizability.

### 4.6. Protein–Protein Interaction Network Construction and Hub Gene Identification

Machine learning-derived feature genes were submitted to the STRING database (version 12.0, https://string-db.org/ (accessed on 19 April 2026)) to construct a PPI network with a confidence score threshold of 0.400 (the default medium-confidence cutoff in STRING, widely used to balance network completeness with interaction reliability) [48]. The network was imported into Cytoscape (version 3.10.1; Institute for Systems Biology, Seattle, WA, USA) [49] for visualization. Hub genes were identified using the CytoHubba plugin by calculating degree, betweenness centrality, and closeness centrality. Genes ranking in the top 10 by at least two of these three metrics were designated hub genes.

### 4.7. Two-Sample Mendelian Randomization Analysis

Two-sample MR was performed to assess causal relationships between genetically predicted expression levels of hub genes and kidney function. The primary exposure instruments were obtained from the eQTLGen Consortium (https://www.eqtlgen.org/ (accessed on 19 April 2026)), which provides blood-based *cis*-eQTL summary statistics from 31,684 individuals [50]. Single-nucleotide polymorphisms (SNPs) significantly associated with hub gene expression ($p<5\times 10^{-8}$) and located within 1 Mb of the gene were selected as instruments. SNPs in linkage disequilibrium (LD, $r^{2}>0.01$) were pruned using the European 1000 Genomes reference panel. As a sensitivity analysis to address potential tissue mismatch between blood and kidney, we additionally extracted *cis*-eQTL data for hub genes from the GTEx v8 kidney cortex dataset (N=73) [37]. Where kidney cortex eQTL instruments reached nominal significance ($p<1\times 10^{-4}$) and contained at least three independent SNPs, supplementary MR analyses were performed to assess directional consistency with blood-based results.

Outcome data for eGFR were obtained from the CKDGen Consortium meta-analysis (N=567,460) [26]. The primary MR method was inverse-variance weighted (IVW) regression. Sensitivity analyses included MR-Egger regression [25] (to assess directional pleiotropy), weighted median estimation (robust when up to 50% of instruments are invalid), and MR-PRESSO [51] (to detect and correct for outlier SNPs). Cochran’s *Q* statistic was used to evaluate heterogeneity. Results were presented as forest plots and scatter plots.

### 4.8. Immune Cell Infiltration Analysis

The CIBERSORT algorithm was applied to the normalized expression matrix of the discovery dataset to estimate the relative proportions of 22 immune cell types in each sample [30]. Samples with a CIBERSORT *p*-value <0.05 were retained for downstream analysis. Differences in immune cell proportions between DKD and control groups were assessed using the Wilcoxon rank-sum test. Spearman correlation coefficients between hub gene expression levels and immune cell fractions were calculated and visualized as a correlation heatmap.

### 4.9. Construction and Evaluation of the Diagnostic Model

A logistic regression-based diagnostic model was constructed using the five hub genes as predictors in the discovery dataset (GSE96804). To mitigate overfitting arising from feature selection and model training on the same dataset, internal validation was performed using 10-fold cross-validation repeated five times; the mean cross-validated AUC (CV-AUC) was reported as the primary measure of internal performance. The final model was then refit on the entire discovery dataset for subsequent external validation. We explicitly acknowledge that the five hub genes used as model predictors were identified through a feature selection procedure applied to the same discovery dataset (GSE96804) prior to this cross-validation loop; consequently, the CV-AUC may be subject to residual optimistic bias from pre-selection. The external validation results in the two independent cohorts (GSE30528 and GSE142025) are therefore presented as the primary and more conservative measures of model generalizability. A fully nested cross-validation design in which feature selection is re-executed within each fold would be required to obtain an unbiased estimate of internal performance, and we recommend this approach in future studies with larger sample sizes. Model discrimination was assessed by the area under the receiver operating characteristic (ROC) curve (AUC) using the pROC package [52]. Calibration was assessed using the Hosmer–Lemeshow goodness-of-fit test. A nomogram was generated with the rms package to provide a visual scoring tool for clinical application. Decision curve analysis (DCA) was performed to evaluate the net clinical benefit of the model across a range of threshold probabilities.

### 4.10. External Dataset Validation

Hub gene expression patterns and diagnostic model performance were validated in two independent GEO datasets (GSE30528 and GSE142025). Expression levels of the five hub genes were compared between DKD and control groups using the Wilcoxon rank-sum test. The logistic regression model trained on GSE96804 was applied to the validation datasets, and AUC values were calculated to assess generalizability.

### 4.11. Drug–Gene Interaction Network Analysis

The Drug–Gene Interaction Database (DGIdb, version 4.0, https://www.dgidb.org/ (accessed on 19 April 2026)) was queried to identify drugs and compounds with known or predicted interactions with the hub genes [53]. Interaction types (inhibitor, agonist, antagonist, etc.) were recorded. The drug–gene interaction network was visualized in Cytoscape.

### 4.12. Molecular Docking Validation

To provide structural-level evidence for potential drug–target interactions, molecular docking was performed between selected candidate compounds and hub gene protein products. Three-dimensional protein structures were retrieved from the Protein Data Bank (PDB, https://www.rcsb.org/ (accessed on 19 April 2026)) or predicted by AlphaFold2. Ligand structures were obtained from PubChem (https://pubchem.ncbi.nlm.nih.gov/ (accessed on 19 April 2026)). Proteins and ligands were prepared using AutoDockTools (version 1.5.7; The Scripps Research Institute, La Jolla, CA, USA): water molecules were removed, polar hydrogens were added, and Gasteiger charges were assigned. Docking was performed using AutoDock Vina (version 1.2.3; The Scripps Research Institute, La Jolla, CA, USA) [54] with a grid box centered on the active site. Binding affinities (kcal/mol) were recorded, and poses with the lowest binding energy were visualized using PyMOL (version 2.5.0; Schrödinger, LLC, New York, NY, USA).

### 4.13. Statistical Analysis

All statistical analyses were performed in R (version 4.3.2). Continuous variables were compared using the Student’s *t*-test (normally distributed) or Wilcoxon rank-sum test (non-normally distributed). Categorical variables were compared using the chi-squared test or Fisher’s exact test. Correlation analyses used Spearman’s rank correlation coefficient. Multiple testing correction was performed using the Benjamini–Hochberg method. A two-sided *p*-value <0.05 was considered statistically significant unless otherwise specified.

## 5. Conclusions

This study identified five hub genes—SPP1, CD44, VCAM1, C3, and TIMP1—as candidate diagnostic biomarkers for DKD through an integrated transcriptomic and machine learning pipeline. Mendelian randomization using blood-derived eQTL instruments provided supportive, but not definitive, evidence for potential causal associations between SPP1, C3, and TIMP1 expression and kidney function decline; these findings require replication using kidney-specific eQTL resources. These genes correlated with pro-inflammatory immune cell infiltration, particularly M1 macrophages, linking them to the inflammatory microenvironment of DKD. A five-gene diagnostic model achieved a cross-validated AUC of 0.938 in the discovery dataset—which may be subject to residual optimistic bias from pre-selection—and AUC values of 0.917 and 0.889 in two independent external validation cohorts. Exploratory drug–gene interaction analysis and molecular docking identified candidate compounds targeting these hub genes, though these predictions require experimental confirmation before any therapeutic relevance can be established. These findings provide a computational framework for generating molecular hypotheses regarding diagnostic biomarkers and potential targeted therapies for DKD; experimental validation—including qPCR, IHC, and prospective clinical cohort studies—is required to confirm these findings, and translation to non-invasive clinical biomarkers requires validation in prospective cohorts using blood or urine samples.

## Figures and Tables

**Table 1:** Hub gene characteristics and centrality metrics in the PPI network.

Gene	log_2_FC	Adj. *p*-Value	Degree	Betweenness	Closeness
SPP1	2.14	$3.2\times 10^{-9}$	7	0.42	0.88
CD44	1.53	$5.7\times 10^{-7}$	6	0.35	0.82
VCAM1	1.48	$8.1\times 10^{-8}$	6	0.31	0.78
C3	1.62	$1.4\times 10^{-7}$	5	0.28	0.75
TIMP1	1.35	$2.9\times 10^{-6}$	5	0.22	0.72

**Table 2:** Two-sample Mendelian randomization results for hub genes and eGFR.

Gene	N SNPs	F-Statistic	IVW β (95% CI)	IVW *p*	Egger Intercept *p*	WM *p*
SPP1	12	187.3	−0.024 (−0.038, −0.010)	$7.8\times 10^{-4}$	0.74	$3.2\times 10^{-3}$
CD44	8	76.5	−0.011 (−0.023, 0.001)	0.067	0.89	0.12
VCAM1	6	42.6	−0.009 (−0.020, 0.002)	0.112	0.65	0.18
C3	15	134.8	−0.019 (−0.031, −0.007)	$2.1\times 10^{-3}$	0.58	$8.7\times 10^{-3}$
TIMP1	10	95.2	−0.016 (−0.028, −0.004)	$8.5\times 10^{-3}$	0.43	0.021

IVW: inverse-variance weighted; WM: weighted median; CI: confidence interval; SNP: single-nucleotide polymorphism.

**Table 3:** GEO datasets used in this study.

Dataset	Platform	Tissue	DKD Samples	Control Samples
GSE96804	GPL570	Glomerular	41	20
GSE30528	GPL570	Glomerular	9	13
GSE142025	GPL17586	Tubulointerstitial	27	9

## Data Availability

All transcriptomic datasets analyzed in this study are publicly available from the NCBI Gene Expression Omnibus (GEO): GSE96804, GSE30528, and GSE142025. eQTL summary statistics are available from the eQTLGen Consortium (https://www.eqtlgen.org/ (accessed on 19 April 2026)). GWAS summary statistics for eGFR are available from the CKDGen Consortium (https://ckdgen.imbi.uni-freiburg.de/ (accessed on 19 April 2026)).

## Abbreviations

The following abbreviations are used in this manuscript:

DKD	Diabetic kidney disease
DM	Diabetes mellitus
CKD	Chronic kidney disease
ESRD	End-stage renal disease
eGFR	Estimated glomerular filtration rate
UACR	Urinary albumin-to-creatinine ratio
GEO	Gene Expression Omnibus
DEG	Differentially expressed gene
WGCNA	Weighted gene co-expression network analysis
LASSO	Least absolute shrinkage and selection operator
RF	Random forest
SVM-RFE	Support vector machine-recursive feature elimination
XGBoost	Extreme gradient boosting
PPI	Protein–protein interaction
MR	Mendelian randomization
IVW	Inverse-variance weighted
SNP	Single-nucleotide polymorphism
eQTL	Expression quantitative trait locus
ROC	Receiver operating characteristic
AUC	Area under the curve
DCA	Decision curve analysis
GO	Gene Ontology
KEGG	Kyoto Encyclopedia of Genes and Genomes
DGIdb	Drug–Gene Interaction Database
